# Antidiabetic Activity of Silver Nanoparticles Biosynthesized with *Stenocereus queretaroensis* Flower Extract

**DOI:** 10.3390/ph18091310

**Published:** 2025-09-01

**Authors:** Angélica Sofía González-Garibay, Iván Moisés Sánchez-Hernández, Omar Ricardo Torres-González, Ana Del Socorro Hernández-Aviña, Ariadna Abigail Villarreal-Amézquita, Eduardo Padilla-Camberos

**Affiliations:** Medical and Pharmaceutical Biotechnology Unit, Center for Research and Assistance in Technology and Design of the State of Jalisco, A.C. (CIATEJ), Av. Normalistas No. 800 Col. Colinas de la Normal, Guadalajara C.P. 44270, Jalisco, Mexico

**Keywords:** silver nanoparticles, diabetes, biosynthesis, *Stenocereus queretaroensis*

## Abstract

**Background/Objectives:** Diabetes mellitus (DM) is one of the most common metabolic disorders, with a continually increasing population incidence. One of the main therapeutic approaches for this condition involves the inhibition of alpha-amylase and alpha-glucosidase—key enzymes involved in carbohydrate breakdown. Silver nanoparticles have exhibited inhibitory activity against both enzymes, suggesting their potential in regulating postprandial blood glucose levels. This study aimed to evaluate the antidiabetic potential of silver nanoparticles biosynthesized with *Stenocereus queretaroensis* flower extract. **Methods:** The flower extract was prepared and, following a qualitative and quantitative phytochemical analysis, was utilized in the reaction to biosynthesize *S. queretaroensis* flower extract nanoparticles (SAgNPs). The SAgNPs were characterized using UV–visible spectroscopy, dynamic light scattering (DLS), scanning electron microscopy (SEM), energy dispersive X-ray (EDX), X-ray diffraction (XRD), and Fourier transform infrared spectrophotometry (FTIR). The antidiabetic potential of the biosynthesized SAgNPs was evaluated in vitro using alpha-amylase and alpha-glucosidase inhibitory assays, while an animal model was used for postprandial hypoglycemic activity in healthy mice. **Results:** The phytochemical analyses showed the presence of phenolic compounds and flavonoids like sinapic acid, p-coumaroyl tyrosine, procyanidin dimer β1, and dihydroquercetin in the flower extract. The SAgNPs were found to be rough and spherical in shape, with an average size of 99.5 nm. The inhibition of alpha-amylase and alpha-glucosidase by SAgNPs exhibited an IC_50_ of 4.92 µg/mL and 0.68 µg/mL, respectively. The animal model results suggested that SAgNPs at 100 mg/kg caused a significant decrease in the postprandial glucose level; this effect is likely attributable to delayed carbohydrate digestion, as supported by the in vitro findings. **Conclusions:** *S. queretaroensis*-synthesized silver nanoparticles may constitute a promising option for antidiabetic therapy.

## 1. Introduction

Diabetes mellitus (DM) affects 537 million adults aged 20 to 79, which represents about 10% of the world’s population, and this number is projected to rise to 643 million by 2030. Further, 90% of people with DM have type 2 diabetes mellitus, according to data from the International Diabetes Federation [1].

DM is characterized by hyperglycemia caused by alterations in insulin secretion and function, which is often associated with one or more diseases, such as cardiovascular disease, liver disorder, or kidney malfunction [2].

Currently, there are different pharmacological treatments for the management of glucose control which act by inhibiting hepatic gluconeogenesis, stimulating insulin production, improving insulin sensitivity, inhibiting glucose reabsorption at the renal level, and decreasing glucose absorption by inhibiting α-amylase and α-glucosidase enzymes [3,4]. However, pharmacological treatments can generate adverse effects, such as lactic acidosis, cardiovascular problems [5], or gastrointestinal discomfort [6], which has led to the search for new therapeutic alternatives with a lower risk of side effects.

In this context, the use of medicinal plants as a complement to pharmacological treatment has gained attention in the scientific community, highlighting bioactive compounds such as flavonoids, tannins, phenols and phenolics, among others [7]. Several studies have proposed the potential of plants to modulate the mechanisms of action involved in glucose regulation [8].

Medicinal plants are also helpful in the field of nanotechnology due to their utility in metallic nanoparticle biosynthesis, mainly for silver. Plant chemical components such as flavonoids can act as silver nitrate-reducing agents and form nanoparticles. It has been documented that some plants high in flavonoids form nanoparticles in a more efficient way [9]. This biological method, known as green synthesis, is cost-effective, energy-efficient, and, most importantly, not hazardous to human health [10,11].

Research on the use of biosynthesized silver nanoparticles in plants for glucose control has shown promising results in combating DM and controlling its symptoms by regulating insulin levels, glucose absorption, and other histochemistry parameters [12,13].

One of the main approaches for hyperglycemic therapies is inhibiting the intestinal enzymes responsible for carbohydrate digestion, such as α-amylase and α-glucosidase, which can, in turn, decrease postprandial blood glucose levels; biogenic silver nanoparticles are thought to be a good fit in this regard [14,15,16,17].

*Stenocereus queretaroensis* is a column-shaped cactus that grows in semi-arid regions of Mexico; its fruit is recognized for its flavor and for its high antioxidant and dietary fiber levels and low caloric content [18]. Infusions of this plant’s flowers have been described as having the ability to control glucose, according to traditional Mexican custom. The chemical composition of *S. queretaroensis* flower extract and its use for the biosynthesis of silver nanoparticles has not yet been reported. The aim of this study is to develop biogenic silver nanoparticles and evaluate their antidiabetic activity in vitro and in a healthy animal model, with a focus on their potential use as co-adjuvant therapies for type 2 diabetes mellitus.

## 2. Results and Discussion

### 2.1. Phytochemical Screening

The phytochemical analysis of the aqueous extract of *S. queretaroensis* flowers (Table 1 and Table 2) focused on the determination of phenolic and flavonoids compounds, as they are considered the main chemical components of botanical extracts involved in the biosynthesis of metal nanoparticles [19]. Our results showed that flavonoids were the main group quantified in flower extract with 202 µg quercetin equivalents/mL.

**Figure 1:**
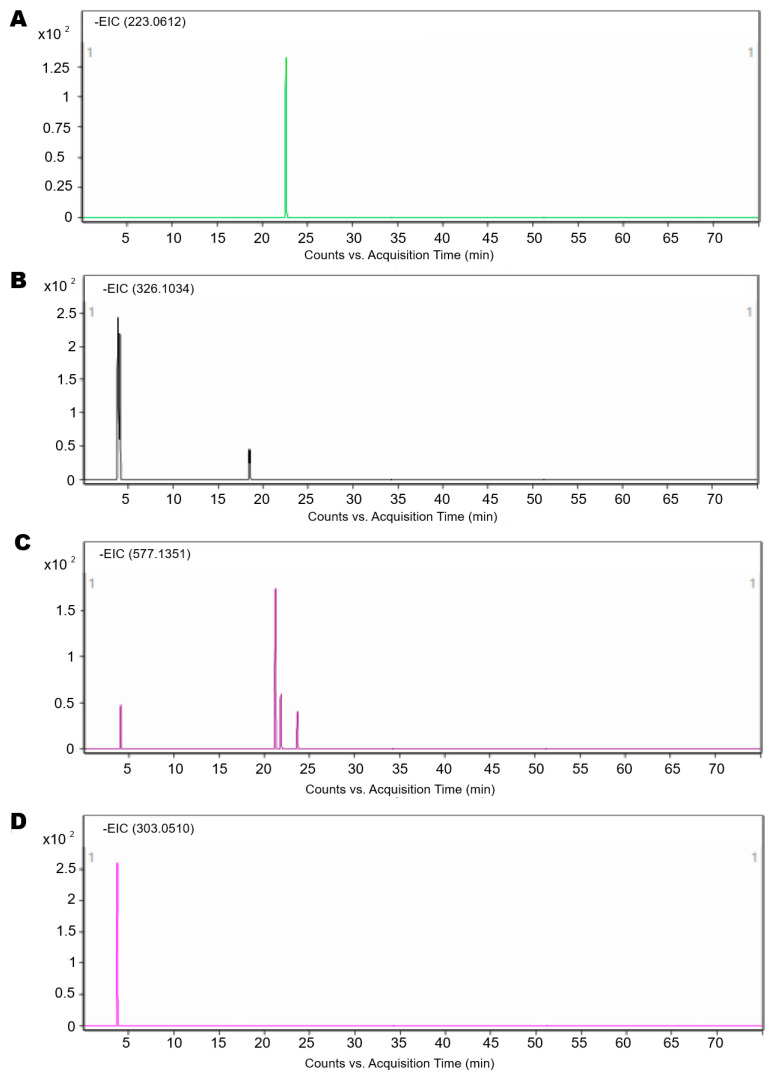
Chromatograms of HPLC-Q-TOF-MS) of *S. queretaroensis* flower extract showing retention time of identified compounds. (**A**) Sinapic acid, (**B**) p-Coumaroyl tyrosine, (**C**) Procyanidin dimer β1, and (**D**) Dihydroquercetin.

The results of high-pressure liquid chromatography coupled with quadrupole time-of-flight mass spectrometry (HPLC-Q-TOF-MS) showed the majority presence of phenolic compounds and flavonoids such as sinapic acid, p-coumaroyl tyrosine, procyanidin dimer β1, and dihydroquercetin, Figure 1. Related compounds such as the flavonoid quercetin and the phenylpropanoid *p*-coumaric acid have also been identified in pitayas of the genus *Stenocereus* [20]. The presence of flavonoids, phenols, hydroxycinnamic acids, and hydroxybenzoic acids in the fruit of *S. queretaroensis* has been previously reported by Noriega-Juárez et al. [18]. However, these findings pertain solely to the fruit and not the flower. To the best of our knowledge, this study presents the first phytochemical characterization of *S. queretaroensis* flower extract.

Some of the compounds present in the extract may serve as reducing and stabilizing agents during nanoparticle synthesis, because they are easily ionized to generate H−ligands and promote the reduction of Ag^+^ to Ag^0^. According to a review of Singh et al., the flavonoids of plant extracts serve as capping agents, which impact on the biological properties of silver nanoparticles [21].

A recent work showed that flavonoids are superior to polyphenols in their role as reducers and stabilizers in nanoparticle biosynthesis [22].

### 2.2. Biosynthesis of Silver Nanoparticles

A color change from light brown to dark brown was observed after 30 min of reaction between *S. queretaroensis* flower extract and AgNO_3_ solution. This color change serves as an indication of silver nanoparticle formation due to the reduction of silver ions in AgNO_3_ [23].

Silver nanoparticle biosynthesis occurred under aqueous *S. queretaroensis* flower extract adjusted to pH 8, mixed in a 1:20 relation ratio with 2 mM silver nitrate solution at 90 °C. These conditions were validated with spectroscopy analyses in the following section.

### 2.3. Ultraviolet−Visible (UV-Vis) Spectroscopy

**Figure 2:**
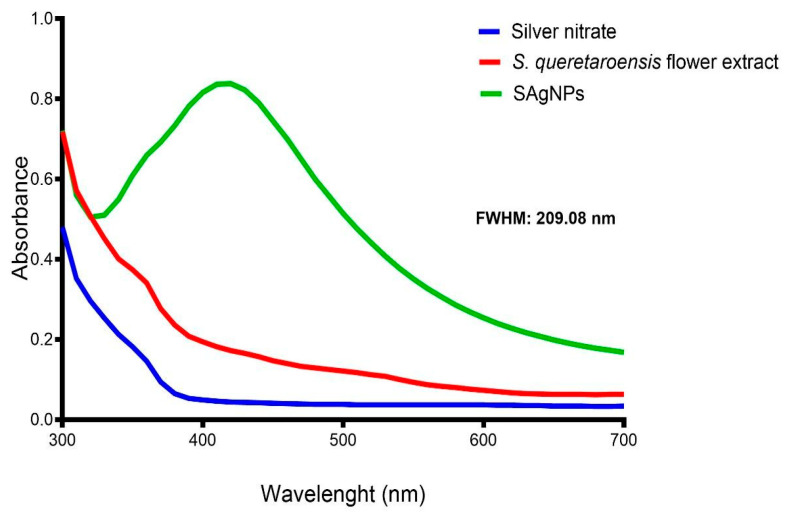
UV–Vis spectra of SAgNPs (green line), aqueous flower extract (red line), and silver nitrate (blue line).

The synthesis of SAgNPs was confirmed via UV–Vis spectroscopy, scanning the reaction product from 300 to 700 nm to observe the plasmon resonance. The UV–Vis absorbance spectrum of SAgNPs revealed a maximum absorbance peak at 420 nm, as previously reported in another study [24]; this wavelength is associated with the conduction of electron oscillation in resonance and confirms the presence of nanoparticles [25]. Neither the flower extract nor the silver nitrate showed absorbance peaks in the analyzed wavelength range, as shown in Figure 2. The full width at half maximum (FWHM) of the spectra is related to the particle size distribution [26] and measured 209.08 nm in SAgNPs.

### 2.4. Particle Size and Zeta Potential

**Figure 3:**
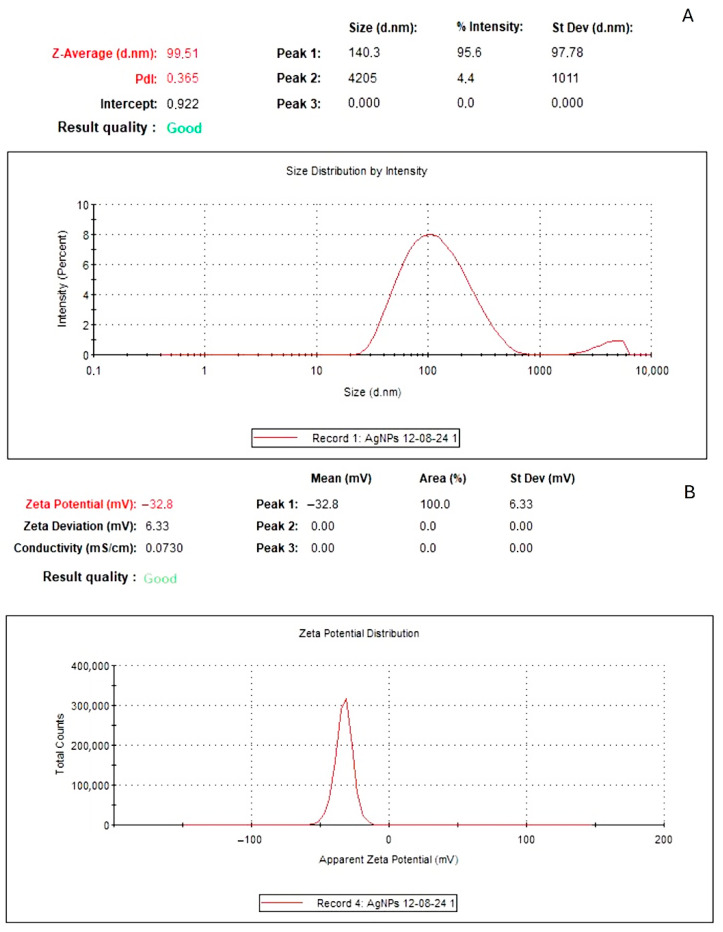
DLS analyses of SAgNPs. Particle size (**A**); zeta potential (**B**).

The particle size of the biosynthesized SAgNPs was determined using dynamic light scattering (DLS). The DLS determines the hydrodynamic size of colloids and can be used to estimate the average nanoparticle size [27]. The results of this study, shown in Figure 3A, indicated that the SAgNPs size was 99.5 nm.

The zeta potential is related to the charges surrounding the nanoparticle surface and has been employed to evaluate the stability of nanoparticles in a solution. Nanoparticles with zeta potentials larger than +30 mV and less than −30 mV show considerable stability in colloidal dispersions [27]. The SAgNPs’ zeta potential, as shown in Figure 3B, was −32.8 mV at pH 7.5. This indicates high stability and denotes that the phytochemicals in the flower extract not only act as reducing agents but stabilize the biosynthesized nanoparticles.

### 2.5. Scanning Electron Microscopy–Energy Dispersive X-Ray Analysis

The morphology and elemental configuration of the SAgNPs were determined by scanning electron microscopy (SEM) with energy dispersive X-ray (EDX) spectroscopy.

**Figure 4:**
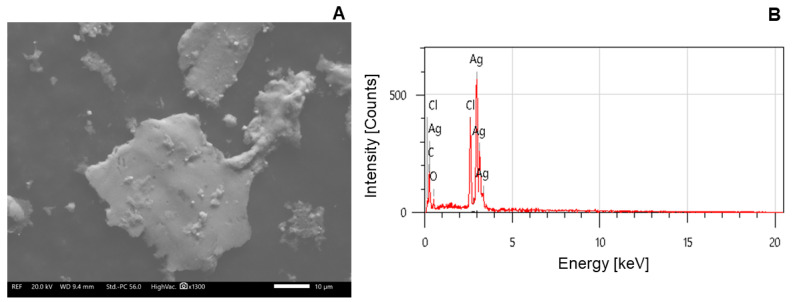
SEM image (**A**) and EDX spectrum (**B**) of SAgNPs.

Based on the SEM images, shown in Figure 4A, the SAgNPs had rough surfaces and were spherical in shape, being less than 10 nm in size. An EDX analysis was conducted to study the SAgNPs’ elemental composition. The EDX spectra, shown in Figure 4B, exhibited an intense peak at 3 keV, confirming that Ag was the main element in the SAgNPs, with a calculated mass of 63.57%. Additionally, the chemical profile showed peaks for chlorine, carbon, and oxygen, in trace amounts, which could be attributed to the phytoconstituents attached to the SAgNPs’ surfaces or the contamination of organic materials and carbon tape used in the measurement process [28]. A similar pattern in the presence of Ag, Cl, O, C, and trace elements has been observed [29].

### 2.6. X-Ray Diffraction Analysis (XRD)

**Figure 5:**
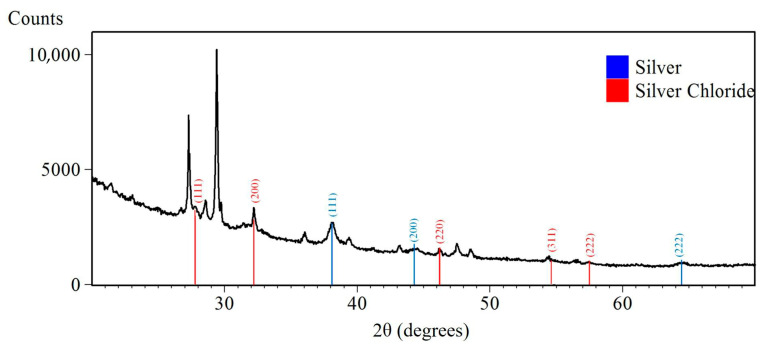
Diffraction peaks from XRD for SAgNPs.

XRD was used to determine the synthesized SAgNPs’ crystalline phase. The XRD spectra of the SAgNPs obtained ranged from 20° to 70°. The X-ray diffraction pattern of the sample exhibits diffraction peaks located at ~38.1°, 44.3°, and 64.5° 2θ, which are indexed to the (111), (200), and (220) crystallographic planes of silver (ICDD: 01-087-0597) (Cifuentes-Jiménez et al., 2024) [30,31]. In addition, the diffraction pattern shows characteristic peaks corresponding to silver chloride (ICDD: 01-087-0597), at ~27.9°, 32.3°, 46.3°, 54.9°, and 57.5° 2θ, attributed to the (111), (200), (220), (311), and (222) planes of AgCl [32,33]; see Figure 5. The phytochemicals *S. queretaroensis* flower extract can act as reducing and stabilizing agents of the SAgNPs, thus providing their crystalline structure, with the latter having been thoroughly studied in various biosynthesized nanoparticles [34]. The crystallite size of the SAgNPs was estimated from the diffractogram using the Debye–Scherrer formula [35] and was found to be 2.07 nm. This size is smaller than that obtained by DLS measurements and SEM analysis, as several crystallites can form a single particle [36].

### 2.7. Fourier Transform Infrared Spectrophotometer Spectroscopy Analysis (FTIR)

**Figure 6:**
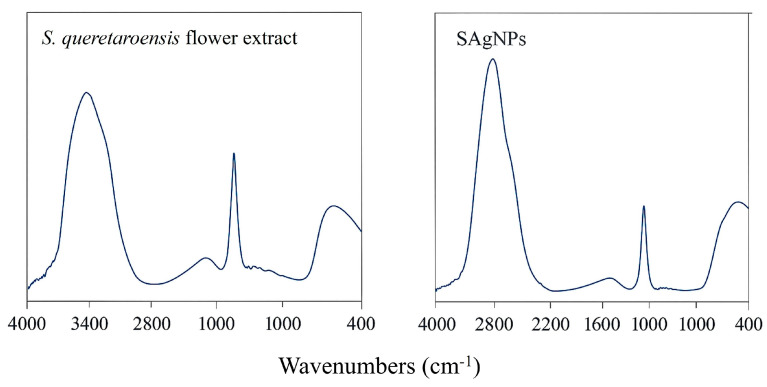
FTIR spectrum of *S. queretaroensis* flower extract (**left**) and SAgNPs (**right**).

FTIR was conducted to determine the possible functional groups responsible for the reduction of Ag^+^. The *S. queretaroensis* flower extract and synthesized SAgNPs were compared at wavenumbers from 4000 to 400 cm^−1^. The infrared spectra of the analyzed samples are presented in Figure 6 and Table 3. The absorption bands at approximately 3451 and 1637 cm^−1^ are attributed to the stretching and bending vibrations of the O–H group, and amides [37], respectively. Additionally, the absorption band at around 2078 cm^−1^ is likely due to the coupling of both vibrational mode types [38].

The SAgNPs’ spectral features are slightly shifted compared to those of the *S. queretaroensis* flower extract. It can be concluded that these phytoconstituents play a significant role in acting as capping agents and enhancing the synthesized nanoparticles’ stability [39].

### 2.8. Antidiabetic Activity In Vitro

Starch digestion in mammals requires multiple enzymes, the most significant of which are α-amylase and α-glucosidase. Together, they rapidly convert digestible starch into glucose. Starch is initially hydrolyzed by human salivary α-amylase in the mouth, followed by human pancreatic α-amylase in the intestine, to produce oligosaccharides. These oligosaccharides are eventually hydrolyzed to absorbable monosaccharides by α-glucosidase. Inhibiting carbohydrate-hydrolyzing enzymes is one of the best approaches for slowing glucose absorption and works by reducing postprandial glucose blood concentration; thus, it represents an important drug target in diabetes prevention and treatment [40,41].

**Figure 7:**
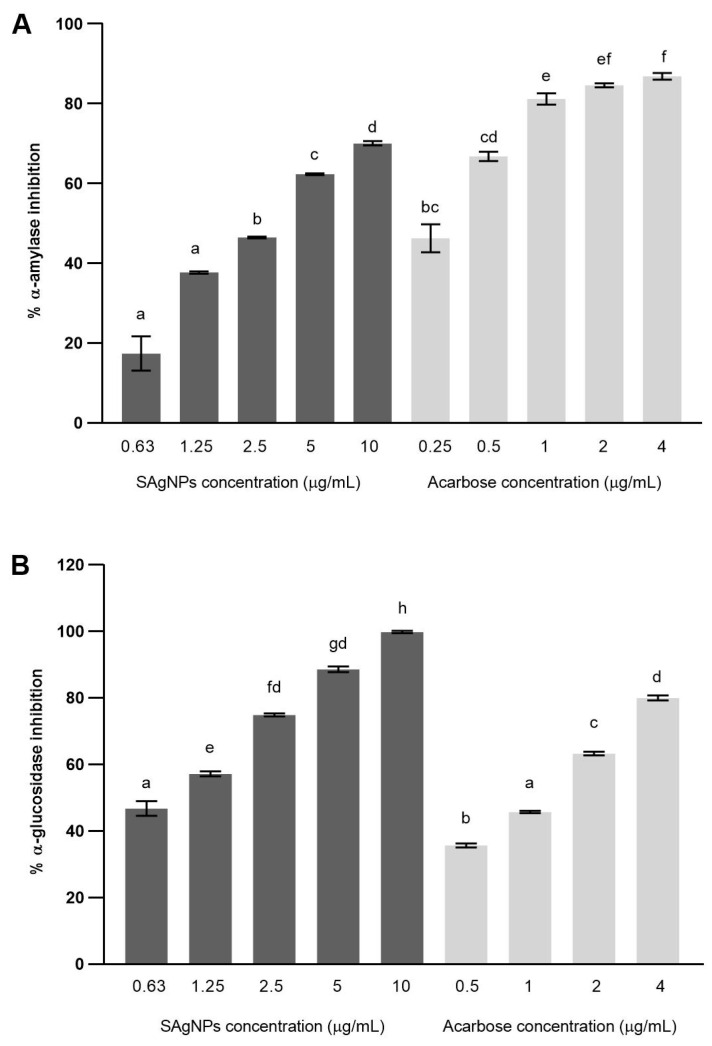
Enzymatic inhibition assay. (**A**) SAgNPs and acarbose on α-amylase inhibition; (**B**) SAgNPs and acarbose on α-glucosidase inhibition. Data are presented as mean ± standard deviation (*n* = 3). Statistical analysis was performed using one-way ANOVA followed by Tukey’s comparison test. Bars with different letters are significantly different (*p* < 0.05).

The SAgNPs inhibit α-amylase and α-glucosidase in a dose-dependent manner, as shown in Figure 7. A maximum of 70.1% inhibition of α-amylase activity was observed at 10 µg/mL, while the maximum inhibition for α-glucosidase was 99.8% at 10 µg/mL.

The SAgNPs showed a slightly higher IC_50_ value for α-amylase, at 4.92 µg/mL (Table 4), suggesting effective inhibitory activity, but with slightly lower potency than that of the standard drug, acarbose. In contrast, the IC_50_ value for α-glucosidase was 0.68 µg/mL, reflecting comparable inhibition to that with acarbose (IC_50_ 1.35 µg/mL). Enzyme inhibitors with lower anti-α-amylase activity and more potent anti-α-glucosidase activity have been proposed for effective postprandial hyperglycemia control with minimal side effects [41]. The possible mechanism of action of SAgNPs could be through competitive inhibition with enzymes, which has been demonstrated in silver nanoparticles synthesized with leaves of *Enicostema axillare* [42].

Several biosynthesized silver nanoparticles have shown inhibitory activity against α-amylase and α-glucosidase at different concentrations. Therefore, it is necessary to consider IC_50_ values in order to be able to make comparisons between studies. A review of the antidiabetic activity of silver nanoparticles synthesized from biological resources indicates IC_50_ values for α-amylase and α-glucosidase inhibition of around 100 μg/mL [43]. However, one study reported IC_50_ values of approximately 16 and 18 μg/mL for α-amylase and α-glucosidase, respectively, using silver nanoparticles derived from *Barringtonia racemosa* leaves [44]. Another study reported a lower IC_50_ value of 0.90 μg/mL against α-amylase using silver nanoparticles synthesized from *Annona muricata* [45], compared to the value obtained in the present work. In contrast, another investigation reported a significantly higher IC_50_ value of 21.51 μg/mL for silver nanoparticles derived from *Ageratum conyzoides* [46], which is nearly four times greater than the value found in this study.

Some authors have proposed that, as a mechanism of action, the chemical constituents of plant extracts attached to silver nanoparticles might attach to the enzyme system and, hence, disrupt enzymatic activity [47]. In fact, flavonoids appear to have better α-glucosidase inhibitory activity than phenolic acids due to the additional hydroxyl groups in the flavone skeleton, which are most likely responsible for flavonoids’ more pronounced inhibitory activity [48].

Regarding glucosidase inhibition by silver nanoparticles, one study reported two types of nanoparticles synthesized from two different kinds of sweet potato, with IC_50_ values of 0.36 and 0.77 μg/mL, respectively [49]. The latter value (0.77 μg/mL) is similar to that obtained in the present study (0.68 μg/mL). In contrast, a more recent study reported an IC_50_ value of 1.68 μg/mL from nanoparticles elaborated with food waste [50], which is more than twice the value observed in this work.

### 2.9. Postprandial Hypoglycemic Activity In Vivo

**Figure 8:**
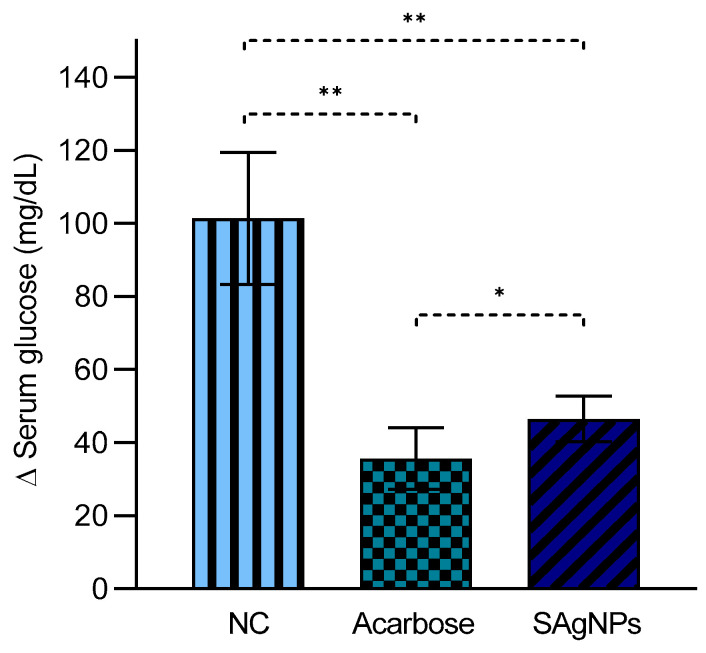
The effect of SAgNPs (100 mg/kg) treatment on fasting blood glucose changes in comparison to the maltose-only (NC) and maltose + acarbose (100 mg/kg) groups. All animals receive a 3000 mg/kg dose of maltose. The data were analyzed using one-way ANOVA following Tukey’s test and are shown as the means ± SEM. *n* = 5 (* *p* < 0.05, ** *p* ≤ 0.01).

The inhibitory effect of SAgNPs on blood sugar levels in vivo was verified by feeding maltose to Balb-c healthy mice. Figure 8 shows the changes in the mouse blood glucose level after feeding them maltose and sampling after 20 min. The results showed that SAgNPs effectively lowered blood glucose levels, although not to the same extent as the positive control acarbose. However, one study utilized a dose twice as high as that of this study in diabetic mice, finding that silver nanoparticles from *Fagonica cretica* lowered blood glucose significantly [16]. The effect of higher doses of SAgNPs requires further exploration. Nevertheless, the results of the animal experiments demonstrated that SAgNPs effectively attenuated the postprandial rise in blood glucose levels, consistent with the findings of the enzyme activity assays.

The antidiabetic activity of biogenic nanoparticles in animal models has been reported by several authors.

For example, one study showed that nanoparticles obtained using a *Thymus serpyllum* aqueous extract improved the insulin sensitivity and glucose tolerance in streptozotocin-induced diabetic BALB/c mice and reduced their fasting glucose levels significantly [13]. On the other hand, the green synthesis of nanoparticles from *Fagonia cretica* leaf extract showed a decrease in biochemical markers, including glucose, in streptozotocin-induced diabetic mice [16].

A previous review has proposed that many characteristics of biogenic silver nanoparticles potentially influence their antidiabetic activity. They can regulate specific gene expressions, inhibit enzymatic activity, and modulate diabetic conditions by radical scavenging. Additionally, they can alleviate diabetes-based conditions by reducing proinflammatory molecules, enhancing wound healing, and influencing additional enzymes to induce antioxidant mechanisms [51]. A meta-analysis using animal models also supported the future potential of silver nanoparticles in antidiabetic applications [52]. Therefore, our SAgNP results correlate well with those in the literature.

A limitation of this study is the absence of an experimental group treated with *S. queretaroensis* flower extract. In the present work, healthy animals with transient hyperglycemia were used to simulate postprandial glucose changes; therefore, it is necessary to explore the effect of SAgNPs in diabetic animals.

## 3. Materials and Methods

### 3.1. Chemical Reagents

All the chemicals used in this study were of analytic grade and purchased from Sigma-Aldrich (Darmstadt, Germany), and distilled water was used for the experiments.

### 3.2. Plant Material

The partially dried *S. queretaroensis* flowers were obtained from a local market. They were immediately washed with distilled water and placed in a Thermolyne I42300 by Thermolyne (Dubuque, IA, USA) convection oven at 60 °C for 24 h. The dehydrated plant material was ground into a fine powder using a porcelain mortar. The powder was stored and protected from light for extraction and further analysis.

#### 3.2.1. Preparation of Aqueous Extract

The dried *S. queretaroensis* flower powder (5 g) was mixed with 250 mL of distilled water at 100 °C for 15 min. The resulting solution was filtered through Whatman No. 1 filter paper and stored at 4 °C until use [53].

#### 3.2.2. Phytochemical Characterization

Qualitative phytochemical analysis was carried out according to the known following standard protocols, and the glycoside content was determined by Fehling’s method [54]. In brief, 0.5 mL of the flower extract was mixed with 1 mL of Fehling’s reagent (CuSO_4_ + NaOH), and the solution was boiled for 5 min. The development of a red color represented the presence of glycosides, while a blue color showed that the solution was negative for these compounds.

The Dragendorff method was used to detect the presence of alkaloids [55]. A volume of 0.1 mL of the aqueous flower extract with 0.9 mL of the Dragendorff reagent (BiNO_3_ + C_4_H_6_O_6_ + KI) was added to a 1.5 mL microtube and allowed to stand for 10 min. The formation of a brownish-red precipitate indicated the presence of alkaloids. Berberine was used as the control.

The Liebermann–Burchard test was used for steroid and triterpenoid determination by dissolving the flower extract in chloroform and acetic anhydride. Two or three drops of sulfuric acid were added to the mixture. During the reaction, the color changed from red at the top of the tube to green, which indicated the presence of sterols, respectively [56].

For the determination of tannins, 1 mL of the flower extract was mixed with 1 mL of 1% ferric chloride. The development of a green or blue color indicated the presence of tannins [57].

Saponins were detected via a foam test by adding 1 mL of the flower extract to 5 mL of distilled water in a test tube. The mixture was vigorously agitated using a vortex for several minutes, with the formation of a persistent foam indicating the presence of saponins [58].

A qualitative phytochemical analysis of flavonoid content was conducted using the aluminum chloride method [59]. In total, 1 mL of flower extract or standard quercetin solution (100 mg/mL) was added to a 10 mL volumetric flask with 4 mL of deionized distilled water (H_2_O). Then, 0.3 mL of 5% NaNO_2_, 0.3 mL of 10% AlCl_3_, and 2 mL of 1M NaOH were added. The solution was made up to 10 mL with H_2_O. A change in coloration (red–orange) was considered indicative of flavonoids. Subsequently, for the quantitative assay, a sample of the final reaction previously obtained was compared to a standard curve of quercetin solution (0–10 mg/mL), and the absorbance was measured at 510 nm. The total flavonoid content was expressed as micrograms of quercetin equivalent per milliliter (μg QE)/mL.

The quantitative phenolic compound content was determined using the Folin–Ciocalteu method [59]. Altogether, 1 mL of the flower extract was added to a volumetric flask with 9 mL of distilled water. Then, 1 mL of the Folin–Ciocalteu reagent (H_3_PMo_12_O_40_ + H_3_PW_12_O_40_) was added and stirred for 5 min. Subsequently, 10 mL of 7% Na_2_CO_3_ was added, and the solution was made up to 25 mL with water, agitating the solution for 90 min at room temperature. A change to a dark color similar to that of gallic acid was considered indicative of the presence of phenols. Subsequently, one sample of the reaction mixture was analyzed and compared to a gallic acid standard curve (0–10 mg/mL) for phenolic quantification. Absorbance was measured at 750 nm, and phenolic content is expressed as micrograms of gallic acid equivalents per milliliter (μg GAE/mL).

Absorbance was measured at 750 nm, and the phenolic content was expressed as micrograms of gallic acid equivalents per milliliter (μg GAE/mL).

#### 3.2.3. Identification of Phenolic Compounds by High-Performance Liquid Chromatography

We performed HPLC-QToF-MS determination using an Agilent 1260-Infinity II liquid chromatograph with a QToF-MS mass detector by Agilent (Santa Clara, CA, USA) and a Phenomenex C_18_ Luna column 5 μm 250 × 4.6 mm (Torrance, CA, USA). The aqueous *S. queretaroensis* flower extract sample was centrifuged and passed through a 0.22 μm Whatman PFTE filter (Marlborough, MA, USA) prior to injection into the equipment.

The extracted ion chromatogram was obtained for each identified compound and searched in the PhytoHub database, version 1.4 [60,61].

### 3.3. Biosynthesis of Nanoparticles

Nanoparticle synthesis was carried out using a method previously reported by our team [62]. Briefly, a 2 mM solution of silver nitrate was prepared and heated; once 90 °C was reached, aqueous *S. queretaroensis* flower extract, prepared as above for phytochemical characterization, and adjusted to pH 8, was added at a 1:20 ratio for a reaction time of 30 min. The SAgNPs obtained were placed in a drying oven at 70 °C for 24 h.

### 3.4. Characterization of SAgNPs

#### 3.4.1. Ultraviolet–Visible Spectroscopy Analysis

The flower extract-mediated reduction of AgNO_3_ to silver nanoparticles was analyzed using an xMark UV–Vis spectrophotometer by Biorad (Hercules, CA, USA). The samples were loaded onto a 48-well microplate and were scanned at a wavelength range of 300 to 700 nm [63]. The FWHM value was obtained from the Gaussian [Ider M] function using Origin software, version 10.2.5.212.

#### 3.4.2. Particle Size and Zeta Potential Measurement

The SAgNPs’ zeta potential and size were determined through DLS with a Zetasizer Nanoseries instrument by Malvern-Panalytical (Malvern, Worcs, UK) to measure the light scattered by particles in the solution. Fluctuations in intensity over time are indicative of the particle size and can be derived using the Stokes–Einstein relation. Zeta potential is the potential at the slipping/shear plane of a colloid particle moving under an electric field. To determine the zeta potential, an electric field is applied, and the electrophoretic mobility of the particles is measured [64].

#### 3.4.3. Scanning Electron Microscopy–Energy Dispersive X-Ray Analysis

The surface morphology and elemental compositions of the synthesized SAgNPs were determined by SEM-EDX, a powerful analytical method widely utilized for surface imaging and elemental analysis at the microscopic scale across diverse scientific disciplines [64]. In this work, a microscope with a fully embedded energy dispersive X-ray spectrometer JEOL JSM-IT800 (Akishima, Tokyo, Japan) was used.

#### 3.4.4. X-Ray Diffraction Analysis

An X-ray diffractogram was obtained in the range of 20–70°2θ with an Empyrean X-ray diffractometer instrument Malvern-Panalytical (Malvern, Worcs, UK) using Bragg–Brentano geometry, a PIXcel1D-Medipix3 detector, and CuKα radiation (λ = 1.5406 Å) at 45 kV and 40 mA. The sample was analyzed with a transmission–reflection spinning station with a size and passage time of 0.0263°2θ and 246.84 s, respectively. The incident beam optics included a 0.04 rad Soller grating, a 10 mm fixed mask, a 1/2° fixed divergence grating, and 2° fixed anti-scatter gratings. On the other hand, a 0.04 rad Soller grating and a 7.5 mm anti-scatter grating were used in the diffracted beam optics, and phases were identified using HighScore Plus software (version 3.0) [65].

#### 3.4.5. Fourier Transform Infrared Spectrophotometer Spectroscopy (FTIR) Analysis

For FTIR analysis, the samples were deposited on KTIR-grade KBr tablets and measured at room temperature using a Nicolet iS10 spectrometer by Thermo Scientific (Waltham, MA, USA) in the range of 4000–400 cm^−1^. In total, 32 spectral analyses were performed on each sample with a resolution of 4 cm^−1^, and the average value was used to obtain the corresponding spectrum to detect the possible functional groups in the biomolecules present in the flower extract [66].

### 3.5. Evaluation of Antidiabetic Activity In Vitro

The α-amylase and α-glucosidase inhibition test for the SAgNPs was performed according to widely reported methods, with modifications.

For the α-amylase inhibition test, 250 µL of SAgNPs (0.63 to 10 µg/mL) or acarbose (0.25 to 4 µg/mL) and 25 µL of a solution of pancreatic α-amylase (0.5 mg/mL) in phosphate-buffer saline (PBS) (0.01 M of phosphate buffer, 0.0027 M of potassium chloride, and 0.137 M of sodium chloride) were added to a 96-well microplate and incubated at 25 °C for 10 min in a shaker. Subsequently, 25 µL of 1% of starch solution in PBS was added and incubated at 25 °C for 10 min, and, finally, 50 µL of dinitro salicylic acid reagent was added and incubated for 5 min at 95–100 °C. After cooling, the absorbance was read at 540 nm on a microplate reader UV-Vis xMark, Bio-Rad (Hercules, CA, USA). Acarbose was used as a control [67].

The α-amylase inhibitory activity is expressed as the percentage inhibition, and it was calculated according to the equation below:

$$\%\;Inhibition=(1-\left(\frac{Sample\;Absorbance}{Negative\;control\;Absorbance}\right))\times 100$$

To determine the enzymatic inhibition of α-glucosidase in a 96-well microplate, 50 µL of SAgNPs (0.63 to 10 µg/mL) or acarbose (0.5 to 4 µg/mL) and 100 µL of α-glucosidase solution (1.0 mg/mL) prepared in PBS were incubated at 25 °C for 10 min. Subsequently, 100 µL of a 0.1 M solution of 4-Nitrophenyl-α-D-glucopyranoside diluted in PBS was added and incubated at 25 °C for 30 min. The absorbance was read at 405 nm at the beginning and at the end of the 5 min incubation period on a microplate reader UV–Vis xMark, Bio-Rad (Hercules, CA, USA) to obtain the absorbance change value. Acarbose was used as the control [68].

The α-glucosidase inhibitory activity was expressed as the percentage inhibition and was calculated according to the following equation:

$$\%\;Inhibition=\left(\frac{Absorbance\;change\;in\;control-Absorbance\;change\;in\;sample}{Absorbance\;change\;in\;control}\right)\times 100$$

### 3.6. Evaluation of Postprandial Hypoglycemic Activity In Vivo

Ethical clearance for the protocol was obtained in strict accordance with the national Mexican Official Norm for Animal Care and Handling (NOM-062-ZOO-1999) [69], classified as category B—experiments that cause minimal discomfort or stress—and was approved by the Internal Committee for Animal Care and Use of Laboratory Animals of Center for Research and Assistance in Technology and Design of the State of Jalisco (approval number 2024-004A).

Regarding toxicological evaluation, our previous studies have demonstrated that nanoparticles biosynthesized using *S. queretaroensis* peel extract do not exhibit acute toxicity following oral, dermal, or inhalation exposure, indicating the absence of adverse effects or mortality. Furthermore, these nanoparticles showed no cytotoxicity toward L929 mouse fibroblasts and did not induce genotoxic or mutagenic effects in vitro models (Padilla-Camberos 2022) [70]. However, those studies were conducted using nanoparticles biosynthesized from the peel extract of *S. queretaroensis*. Therefore, further investigations are necessary to thoroughly assess the safety and potential risks of SAgNPs biosynthesized using the flower extract.

Healthy mice (15 animals, Balb-c strain, 8 weeks old), acquired by Bioterio Morelos, were housed in an animal room at 20−22 °C with a 12 h light/dark cycle. According to the health certificate from the supplier, the animals were healthy, and there were no exclusions. After 5 days of acclimation-free access to water and food, they were randomly divided into three groups (*n* = 5 for each group, according to previous studies) [71]. This included a control group (maltose 3000 mg/kg), a positive group (maltose + acarbose 300 mg/kg), and a SAgNPs group (maltose + SAgNPs 100 mg/kg). The dose used was selected according to previous studies [72,73].

After fasting for 12 h, all animal groups were gavaged with maltose solution (3000 mg/kg), with each treated animal being identified by a mark. At the same time, samples were administered intragastrically, and the initial glucose level was measured in the tail vein using a One Touch, Lifescan (Paterson, NJ, USA) portable glucometer. After 20 min of gavage, the glucose level was measured again [74].

### 3.7. Statistical Analysis

The data are expressed as the mean ± standard error of the mean. Statistical analysis was performed using GraphPad Prism version 8.0.1. One-way analysis of variance (ANOVA) was employed, followed by Tukey’s post hoc test. Differences were considered statistically significant at *p* < 0.05.

## 4. Conclusions

In conclusion, the current study revealed that silver nanoparticles can be synthesized using a biological method employing *S. queretaroensis* flower extract, a plant popularly used for diabetes management in traditional medicine. To the best of our knowledge, this is the first study to report the phytochemical analysis of *S. queretaroensis* flower extract, revealing the presence of active phytoconstituents such as phenolic and flavonoid compounds. The SAgNPs were characterized using UV−Vis spectroscopy, DLS, SEM-EDX, XRD, and FTIR. The biosynthesized SAgNPs measured 99.5 nm in size.

In the present study, we established that SAgNPs display α-amylase- and α-glucosidase-inhibiting activity in vitro. SAgNPS reduced postprandial glucose level in healthy animals, an effect likely attributable to delayed carbohydrate digestion, as supported by the in vitro findings. It is further recommended that the mode of action of these nanoparticles should be studied in preclinical studies with diabetic animals. Further research into the pharmacokinetics and safety of SAgNPs is necessary to develop nanodrugs for glycemic management in people with type 2 diabetes mellitus, which is most prevalent in the population.

## Figures and Tables

**Table 1:** Qualitative phytochemical analysis in flower extract. Present (+); absent (−).

Qualitative Analysis	
Glucosides	−
Alkaloids	−
Triterpenoids	−
Tannins	+
Sterols	+
Saponins	+
Phenolic compounds	+
Flavonoids	+

**Table 2:** Quantitative phytochemical analysis in flower extract (GAE, gallic acid equivalents; QE, quercetin equivalents).

Quantitative Analysis	
Phenolic compounds	172.81 µg GAE/mL
Flavonoids	202 µg QE/mL

**Table 3:** Peak positions related to functional groups in FTIR analysis.

Sample	Absorption Band	Functional Group
*S. queretaroensis* flower extract and SAgNPs	3451	O-H
	1637	Amide
	2078	Coupling

**Table 4:** IC_50_ values of SAgNPs and acarbose for α-amylase and α-glucosidase inhibition.

	IC_50_ (µg/mL)	
Sample	Enzymes	
	α-amylase	α-glucosidase
SAgNPs	4.92	0.68
Acarbose	1.38	1.35

## Data Availability

The data are contained within the article.
